# How movies move us – movie preferences are linked to differences in neuronal emotion processing of fear and anger: an fMRI study

**DOI:** 10.3389/fnbeh.2024.1396811

**Published:** 2024-06-04

**Authors:** Esther Zwiky, Philine König, Rebekka Maria Herrmann, Antonia Küttner, Janine Selle, Lena Esther Ptasczynski, Konrad Schöniger, Mareike Rutenkröger, Verena Enneking, Tiana Borgers, Melissa Klug, Katharina Dohm, Elisabeth J Leehr, Jochen Bauer, Udo Dannlowski, Ronny Redlich

**Affiliations:** ^1^Department of Psychology, Martin-Luther-University Halle-Wittenberg, Halle (Saale), Germany; ^2^German Center for Mental Health (DZPG), Site Jena-Magdeburg-Halle, Halle (Saale), Germany; ^3^Department of Psychiatry and Neurosciences, Charité –Universitätsmedizin Berlin, Berlin, Germany; ^4^Berlin School of Mind and Brain, Humboldt University of Berlin, Berlin, Germany; ^5^Department of Medical Psychology, University Medical Center Hamburg-Eppendorf, Hamburg, Germany; ^6^Institute for Translational Psychiatry, University of Münster, Münster, Germany; ^7^Department of Radiology, University of Münster, Münster, Germany; ^8^Center for Intervention and Research on Adaptive and Maladaptive Brain Circuits Underlying Mental Health (C-I-R-C), Jena-Magdeburg-Halle, Halle (Saale), Germany

**Keywords:** emotion processing, movie preferences, amygdala, nucleus accumbens, functional magnetic resonance imaging, fMRI, fear, anger

## Abstract

**Introduction:**

As a source of audio-visual stimulation, movies expose people to various emotions. Interestingly, several genres are characterized by negative emotional content. Albeit theoretical approaches exist, little is known about preferences for specific movie genres and the neuronal processing of negative emotions.

**Methods:**

We investigated associations between movie genre preference and limbic and reward-related brain reactivity to close this gap by employing an fMRI paradigm with negative emotional faces in 257 healthy participants. We compared the functional activity of the amygdala and the nucleus accumbens (NAcc) between individuals with a preference for a particular movie genre and those without such preference.

**Results and discussion:**

Amygdala activation was relatively higher in individuals with action movie preference (*p*_TFCE-FWE_ = 0.013). Comedy genre preference was associated with increased amygdala (*p*_TFCE-FWE_ = 0.038) and NAcc activity (*p*_TFCE-FWE_ = 0.011). In contrast, crime/thriller preference (amygdala: *p*_TFCE-FWE_ ≤ 0.010, NAcc: *p*_TFCE-FWE_ = 0.036), as well as documentary preference, was linked to the decreased amygdala (*p*_TFCE-FWE_ = 0.012) and NAcc activity (*p*_TFCE-FWE_ = 0.015). The study revealed associations between participants’ genre preferences and brain reactivity to negative affective stimuli. Interestingly, preferences for genres with similar emotion profiles (action, crime/thriller) were associated with oppositely directed neural activity. Potential links between brain reactivity and susceptibility to different movie-related gratifications are discussed.

## 1 Introduction

Since its beginning in the late 19th century, movies have remained a central source of entertainment. In 2019, the global theatrical and home entertainment market surpassed the $100 billion mark (Escandon, 2020) and currently, the two most commercially successful streaming platforms, Netflix and Amazon Prime Video have over 400 million members worldwide (Digital TV Research, 2022; Netflix, 2023). The question of what exactly enthralls and drives people to watch movies has occupied researchers in various fields since early on. In his pioneer work, The Photoplay: A Psychology Study, Münsterberg (1916) states that the central aim of creating movies lies in picturing emotions (p. 112). To this day, various scholars share Munsterberg’s opinion and highlight the emotional experience as the key to keeping the audience absorbed and entertained by movies (Tan, 1996, 2018; Smith, 2003). Moreover, emotions elicited by movies and those triggered by real-life events have shown similarities on a physiological (e.g., electrodermal activity and heart rate, Hubert and De Jong-Meyer, 1991); expressional (e.g., facial expressions, Zempelin et al., 2021), neuronal (e.g., fear, Hudson et al., 2020) and behavioral level (e.g., startle response, Koukounas and McCabe, 2001); freezing response, Hagenaars et al., 2014). Furthermore, movies have been proven to induce emotional states effectively in experimental settings (Fernández-Aguilar et al., 2019).

However, qualitative and quantitative differences in emotions displayed and elicited by movies exist within the spectrum of genres. While certain genres are known for their lighthearted watching experience and thus their positive affective valence (e.g., comedy, romance), others predominantly convey emotions of negative valence (e.g., horror, thriller). Depending on the density of emotional cues (e.g., cut frequency, audio effects, facial expressions), some genres are more likely to transport intense emotions than others (Smith, 2003). Apart from the valence of emotions, movies also differ regarding the arousal level they might induce in viewers. While various genres picture or elicit high-arousing emotions, like fear and anger, others might promote a low-arousing experience by depicting nature sceneries (e.g., nature documentaries) or emotions associated with more internalizing, withdrawal behavior like sadness in dramas. Furthermore, movie genres are usually linked to one or two dominant discrete emotions (Grodal, 2017). Some (e.g., action, crime/thriller) primarily trigger early evolved emotions (e.g., fear, anger, seeking), which are associated with ancient brain parts like the brainstem and the amygdala. Others (e.g., drama, romance, comedy) trigger more recently developed mammalian and social emotions (e.g., care, grief, play), predominately linked to neocortical functions (Grodal, 2017). Some genres, like documentary, are usually not linked to any emotional qualities due to their factual nature.

Interestingly, genres primarily picturing and aiming to elicit negative emotions are extremely popular and attract broad audiences. The alleged paradox of people voluntarily seeking emotions in movie entertainment, usually experienced as a reaction to aversive real-life events, has been broadly discussed (e.g., Oliver, 1993; Hanich, 2010; Wagner et al., 2014; Menninghaus et al., 2017). Some researchers apply the concept of meta-emotions to unravel this phenomenon (Oliver, 1993; Bartsch, 2007a; Bartsch et al., 2008). Meta-emotions result from the appraisal of primary emotions and are a motivational force in maintaining or changing primary emotions (Bartsch et al., 2008). If the appraisal of the primary emotion is favorable, positive meta-emotions (e.g., pleasure, enjoyment) arise and motivate to continue turning to the primary emotions. Therefore, if followed by positive meta-emotions, even unpleasant emotions could appear rewarding. Several studies investigated the impact of meta-emotions on the evaluation of audiovisual content. Positive meta-emotions in reaction to music videos incorporating either horror or drama elements were associated with positive evaluations of those music videos and preferences for horror and drama movies, respectively (Bartsch, 2007b). Hanich et al. (2014) demonstrated that the feeling of being moved (positive meta-emotion) mediates the positive evaluation of sad movies. By predicting the evaluation of movie content, meta-emotions could be the missing link between negative movie-elicited emotions and preferences for negative emotion-predominant genres. Thus, Bartsch (2007a) refers to genre preferences as action tendencies stemming from meta-emotions. Menninghaus et al. (2019) also state that positive aesthetic emotions (e.g., suspense, joy, excitement) are often promoted by negative art-elicited emotions. Positive aesthetic emotions, in turn, predict approaching certain art content and, ultimately, genre preference formation (Menninghaus et al., 2019). Consequently, depending on whether and to which extent a valence switch is happening from a distinct negative emotion elicited by a movie (e.g., fear, anger, sadness) to a positive meta-emotion (e.g., enjoyment), individuals are drawn to movie genres characterized by this distinct emotion or not.

According to Grodal (2017), movie genres are cultural constructs influenced by the brain’s preferences and emotional and action potentials. In other words, the human brain’s reaction tendencies determine movie preferences. Differences in (neuro) biology should, therefore, predict differences in preferences for specific movie content and genres. Conversely, movie genres are seen as social constructs based on shared (neuro) biological predispositions. However, besides age, sex, and partly biologically determined personality traits, little is known about (neuro) biological correlates of movie genre preferences. Since emotions are considered a vital element of the entertaining experience of movie watching and a crucial source of gratification, neurobiological correlates of emotional processing may play a role in differences in movie genre preference. Neuroimaging research has shown that different movie-elicited emotions like fear (emotional peak) and suspense (anticipation of fear) are linked to dissociable patterns of neuronal activity (Hudson et al., 2020). For poetry or music, there is also evidence that the anticipation (Wassiliwizky et al., 2017) or experience (Salimpoor et al., 2011) of chills, as an expression of emotional arousal, is correlated with increased nucleus accumbens (NAcc) activity as a central structure of the neuronal reward system. However, there are no studies that investigated the preference for specific movie genres and brain functional associations towards emotional stimuli.

To close this gap, the present study investigates whether preferences for specific movie genres of distinct emotional profiles are linked to differences in brain activity during emotional processing using functional magnetic resonance imaging (fMRI). Given the high popularity and broad range of genres that trigger negative emotions, the present study focuses on genre-related differences in brain activity while processing material of negative emotional valence. Due to the lack of studies focusing on neurobiological correlates of movie genre preference, strong *a priori* hypotheses are challenging. However, because of different dispositions leading to particular movie preferences, or as a result of repetitive watching of movies with a genre-specific emotional profile or an interplay of both, differences in reactivity in the amygdala as a central structure of processing primary/basic emotions, like fear and anger, are hypothesized. In line with former research, we argue that movie-elicited negative emotions could be a central factor for movie enjoyment and thus play a crucial role, especially in preferencing genres that predominantly picture and elicit negative emotions. Consequently, genre preference-dependent variations in the activity of neural reward circuits, especially the NAcc, are expected to indicate variances in the approach tendency towards fearful and angry faces.

## 2 Materials and methods

### 2.1 Participants and study design

The study was part of the larger ongoing Muenster (Germany) Neuroimaging Cohort study investigating the neurobiology of affective disorders. In order to investigate potential associations between movie preference and limbic reactivity, data from 275 participants was acquired. Due to missing data or violation of choice restrictions, 18 participants were excluded, resulting in a final sample size of *N* = 257 (age: *M* = 39.95, SD = 13.26; sex (m/f): 129/128). Participants were recruited through public notices and newspaper announcements. For more detailed sample characteristics, see Table 1.

**Table 1:** Sociodemographic sample and group characteristics.

Characteristic	Full sample	Action	Crime/ thriller	Drama	Romance	Comedy	Documentary	Sci-fi/ fantasy
	(*N* = 257)	(*n* = 58)	(*n* = 114)	(*n* = 31)	(*n* = 37)	(*n* = 79)	(*n* = 111)	(*n* = 39)
	*M (SD)*	*M (SD)*	*M (SD)*	*M (SD)*	*M (SD)*	*M (SD)*	*M (SD)*	*M (SD)*
Age (years)	39.95 (13.26)	36.57 (11.68)	42.66 (13.17)	33.13 (12.08)	38.41 (13.90)	37.35 (13.63)	44.86 (12.63)	33.41 (10.47)
Sex (m/f)	129/128	41/17	58/56	14/17	0/37	36/43	52/59	29/10
Daily media consumption time (min)	99.00 (73.69)	98.36 (91.71)	111.54 (73.02)	96.19 (74.50)	128.65 (130.58)	97.03 (55.79)	92.40 (55.47)	89.38 (61.53)
Verbal-IQ (MWTB)	114.63 (12.44)	110.64 (10.66)	116.87 (12.08)	112.13 (10.55)	111.86 (11.81)	114.27 (12.89)	116.13 (13.78)	114.92 (9.77)
Highest education degree (%)								
Degree after 8–9 school years	3.5	3.4	2.6	3.2	2.7	1.3	5.4	2.6
Secondary school diploma (10 years)	14.0	12.1	14.0	9.7	13.5	16.5	16.2	10.3
Vocational diploma (11 school years)	7.4	6.9	8.8	16.1	2.7	8.9	5.4	5.1
High school degree	21.0	15.5	21.1	22.6	16.2	32.9	15.3	28.2
Apprenticeship	17.9	24.1	15.8	12.9	32.4	8.9	20.7	7.7
Master craftsman	3.1	3.4	6.1	0.0	0.0	3.8	0.9	2.6
Bachelor’s degree	7.4	12.1	5.3	12.9	13.5	6.3	4.5	12.8
Master’s degree	25.7	22.4	26.3	22.6	18.9	21.5	31.5	30.8
Housing situation (%)								
Living alone	16.7	17.2	15.8	19.4	18.9	16.5	12.6	23.1
With romantic partner (unmarried)	11.7	10.3	9.6	29.0	8.1	7.6	10.8	17.9
With spouse	48.6	46.6	57.0	19.4	45.9	48.1	55.9	41.0
With parents/relatives	9.3	12.1	5.3	12.9	8.1	15.2	8.1	7.7
Dormitory/shared apartment	12.1	12.1	12.3	19.4	16.2	12.7	9.9	7.7
Other	1.6	1.7	0.0	0.0	2.7	0.0	2.7	2.6

Exclusion criteria were any neurological abnormalities or traumatic head injuries, organic mental disorders, dementia, chronic medical diseases, any lifetime psychiatric disorders according to the SCID-I (Wittchen et al., 1997), or MRI contraindications. All participants were free of psychotropic medication, had a normal or corrected-to-normal vision, and their verbal IQ, estimated with a German multiple-choice vocabulary intelligence test (MWT-B; Lehrl et al., 2005), was within one standard deviation (SD = ± 15) or above. Participants received an assessment of movie preferences and sociodemographic variables prior to the fMRI session. The experimental procedure was approved by the local Institutional Review Board (IRB). All participants gave written informed consent and received financial compensation.

### 2.2 Assessment of movie preferences

To assess movie preferences and media consumption, participants were asked to select their preference out of eight movie genres (action, crime/thriller, horror, drama, romance, comedy, documentary, science-fiction/fantasy). To receive a realistic picture of individuals’ movie preferences and to prevent potential information loss due to a forced single choice, participants had the option to select two equally preferred genres, which 84.8% used. Note that individuals were assigned to two groups in case two preferred genres were reported. Due to the small sample size (*n* = 6), the horror genre was excluded from all analyses, leaving seven movie genres (action, crime/thriller, drama, romance, comedy, documentary, science-fiction/fantasy) for fMRI analyses. For group characteristics, see Table 1.

### 2.3 Paradigm

To investigate neural emotional/limbic reactivity, a frequently used paradigm for eliciting amygdala responsiveness was applied as an experimental fMRI paradigm (Hariri et al., 2002; Dannlowski et al., 2012; Redlich et al., 2015b; Enneking et al., 2020). The face-matching task consisted of four blocks of a face-processing task and five blocks of a sensorimotor control task. The face-processing task showed three faces expressing anger or fear. Participants should choose one of two faces from the bottom of the screen that was identical to the third face on the top. Each block of the face-processing task consisted of six trials, balanced for emotion (fear, anger) and target sex taken from the Ekman stimulus set (Ekman, 1976). The sensorimotor control task showed three geometric shapes (circles and ellipses). Participants were instructed to select one of the two shapes on the bottom identical to the shape on the top. Each sensorimotor control block consisted of six shape trios. All blocks were preceded by an instruction inviting the participants to match either faces or shapes shown for 2 sec. In the face processing blocks, face trios were presented for 4 sec with a variable inter-stimulus interval (1.5–5.5 s, *M* = 3.5 s), resulting in a total block length of 47 s. The six shape trios of the sensorimotor control blocks were presented for 4 sec each with a fixed inter-stimulus interval of 1.5 s, adding up to a total block length of 35 s. The total task time was 363 s.

### 2.4 Functional MRI data acquisition and preprocessing

For functional MRI methods and statistical approach, we followed well-established protocols (Opel et al., 2015; Redlich et al., 2015a,b,c). Concisely, T2* functional data were acquired with a 3 Tesla scanner (Gyroscan Intera 3 T, Philips Medical Systems, Best, NL) using a single-shot echoplanar sequence, with parameters selected to minimize distortion in the region of central interest while retaining an adequate signal-to-noise ratio (S/N) and T2* sensitivity. Volumes consisting of 34 slices were acquired (matrix 64×64, resolution 3.6 mm × 3.6 mm × 3.6 mm; TR = 2.1 s, TE = 30 msec, FA = 90°). To minimize drop-out artifacts in the mediotemporal and orbitofrontal regions, slices were tilted by 25° from the AC/PC line. The stimuli presentation was projected to the rear end of the scanner (Sharp XG-PC10XE with additional HF shielding). Data preprocessing was performed using statistical parametric mapping software (SPM8, Welcome Department of Cognitive Neurology, London, United Kingdom; http://www.fil.ion.ucl.ac.uk/spm), including realignment, unwarping, and spatial normalization of each participant’s functional images to the Montreal Neurological Institute International Consortium (MNI) for Brain Mapping template. Images were smoothed with a Gaussian kernel of 6 mm full-width at half-maximum (FWHM).

### 2.5 Statistical analyses

Statistical analyses of fMRI data were carried out using statistical parametric mapping software (SPM12, Welcome Department of Cognitive Neurology, London, United Kingdom; http://www.fil.ion.ucl.ac.uk/spm). Sociodemographic and movie preference data were analyzed using SPSS Statistics (version 27.0; IBM Corporation).

#### 2.5.1 First-level analyses

The onsets of the experimental conditions (faces, shapes) were modeled using a canonical hemodynamic response function in the context of a general linear model. Low-frequency noise was removed by applying a high-pass filter of 128 s. An individual contrast image was generated in each first-level analysis (faces > shapes).

#### 2.5.2 Second-level analyses

For each movie genre a two-sample *t*-test of the contrast images (faces > shapes) was performed in order to investigate differences between participants with and without preference for the particular movie genre (*preference* vs. *no preference*). Considering previously reported correlations with movie genre preference, age, and sex were included as covariates of no interest in all models, as well as the average daily media consumption time (*time*) to control for possible general exposure effects. According to our hypotheses, ROI analyses of the bilateral amygdala and NAcc were performed separately. ROIs were created by means of the Wake Forest University PickAtlas (Maldjian et al., 2003) according to the AAL-atlas definitions (Tzourio-Mazoyer et al., 2002) for the amygdala and IBASPM 71 atlas definitions (Alemán-Gómez et al., 2006) for the NAcc. For ROI analyses, significance thresholds for multiple testing were obtained at the cluster level by threshold-free cluster enhancement (TFCE), implemented in the TFCE toolbox (http://dbm.neuro.uni-jena.de/tfce, version 232). A conservative family-wise error (FWE)-corrected threshold of *p* < 0.05 was established, obtained by 5,000 permutations per test.

## 3 Results

### 3.1 Group differences between preference vs. no preference

**Figure 1:**
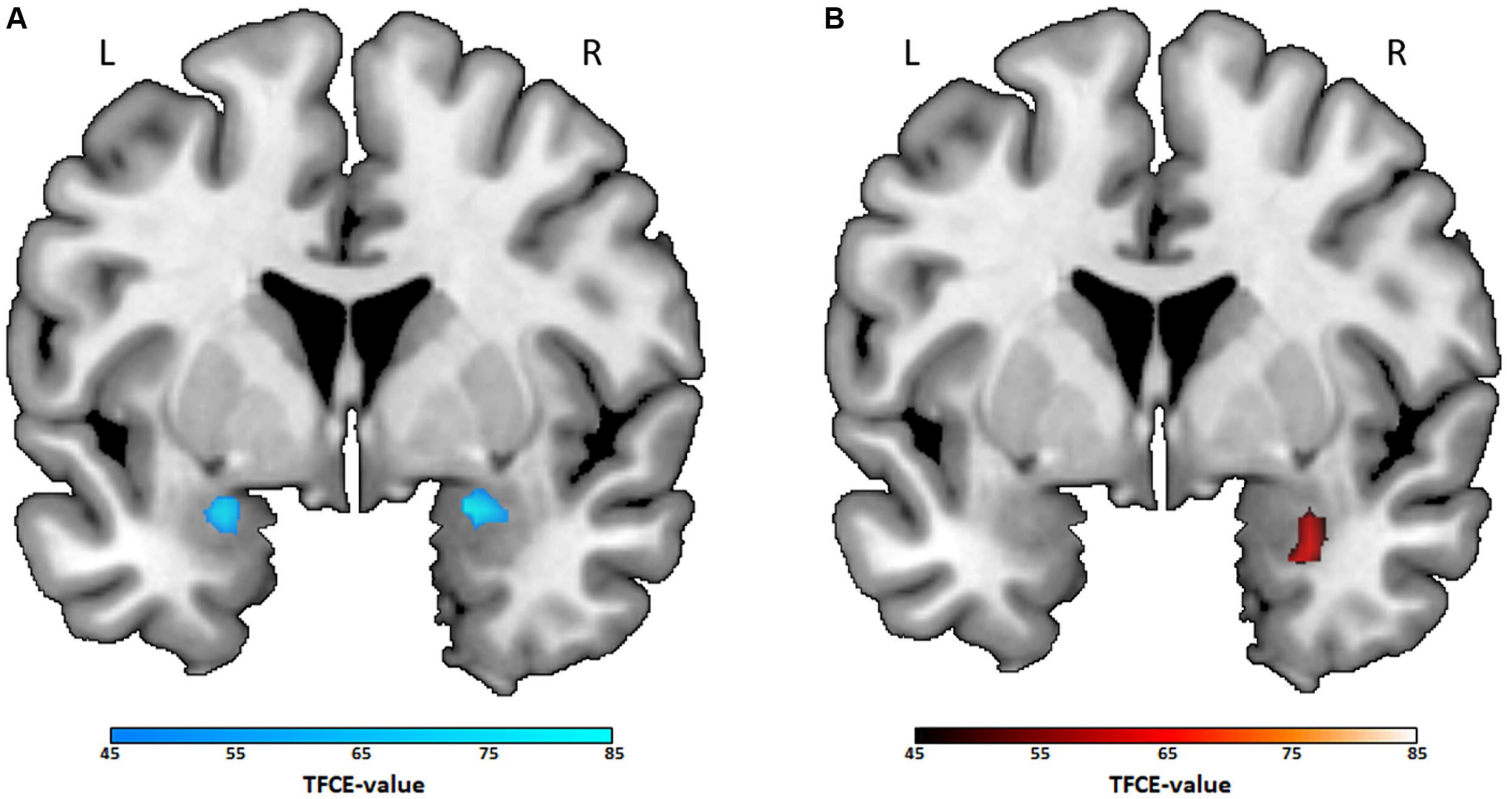
Differences in amygdala activity between preference and no preference groups for crime/thriller and action. Note. Coronal views of the ROI analyses of the amygdala (*y* = 2) for two-sample *t*-tests including age, sex, and time as covariates (*p*_TFCE-FWE_ < 0.05). Color bar: TFCE-value. **(A)** Contrast crime/thriller preference < no crime/thriller preference (mean cluster value at: left: *x* = −24, *y* = 2, *z* = −18, *k* = 76; right: *x* = 24, *y* = 2, *z* = −18, *k* = 68). **(B)** Contrast action preference > no action preference (mean cluster value at: *x* = 30, *y* = 4, *z* = −26, *k* = 58). L, left hemisphere; R, right hemisphere.

The ROI analyses showed significant group differences in limbic reactivity to emotional stimuli for four genres (crime/thriller, documentary, action, comedy). The analysis of crime/thriller revealed bilateral effects in the amygdala, resulting in decreased activity during negative face processing for the preference group compared to the no preference group (left: *x* = −24, *y* = 2, *z* = −18, *k* = 76, TFCE_(252)_ = 71.49, *t* = 3.17, *p*_TFCE-FWE_ = 0.010; right: *x* = 24, *y* = 2, *z* = −18, *k* = 68, TFCE_(252)_ = 78.31, *t* = 3.42, *p*_TFCE-FWE_ = 0.008, see Figure 1A). Furthermore, unilateral differences in the right amygdala occurred for documentary, action, and comedy genres. While the documentary preference group showed decreased amygdala activity (*x* = 32, *y* = 4, *z* = −26, *k* = 47, TFCE_(252)_ = 65.49, *t* = 3.25, *p*_TFCE-FWE_ = 0.012), activity was increased for the action genre preference group (*x* = 30, *y* = 4, *z* = −26, *k* = 58, TFCE_(252)_ = 66.68, *t* = 3.26, *p*_TFCE-FWE_ = 0.013, see Figure 1B) and the comedy preference group (*x* = 26, *y* = −2, *z* = −18, *k* = 17, TFCE_(252)_ = 45.25, *t* = 2.78, *p*_TFCE-FWE_ = 0.038), each compared to the no preference group, respectively. For drama, romance, and science-fiction/fantasy, the ROI analyses of the bilateral amygdala showed no significant group differences (preference vs. no preference, all *p*_TFCE-FWE_ ≥ 0.166).

The ROI analyses of the NAcc showed significant right-sided group differences in activity of neural reward circuits to emotional stimuli for three genres: crime/thriller (*x* = 14, *y* = 12, *z* = −10, *k* = 5, TFCE_(252)_ = 10.26, *t* = 2.57, *p*_TFCE-FWE_ = 0.036), documentary (*x* = 10, *y* = 12, *z* = −10, *k* = 3, TFCE_(252)_ = 11.98, *t* = 2.77, *p*_TFCE-FWE_ = 0.015) and comedy (*x* = 12, *y* = 12, *z* = −10, *k* = 5, TFCE_(252)_ = 11.76, *t* = 2.60, *p*_TFCE-FWE_ = 0.011). While the crime/thriller and the documentary preference groups showed decreased activity in the NAcc, activity was increased in the comedy genre group compared to the no preference group. For action, drama, romance, and science-fiction/fantasy, the ROI analyses of the NAcc showed no significant group differences (preference vs. no preference, all *p*_TFCE-FWE_ ≥ 0.106).

### 3.2 *Post-hoc*: direct comparisons of different genre preferences

**Figure 2:**
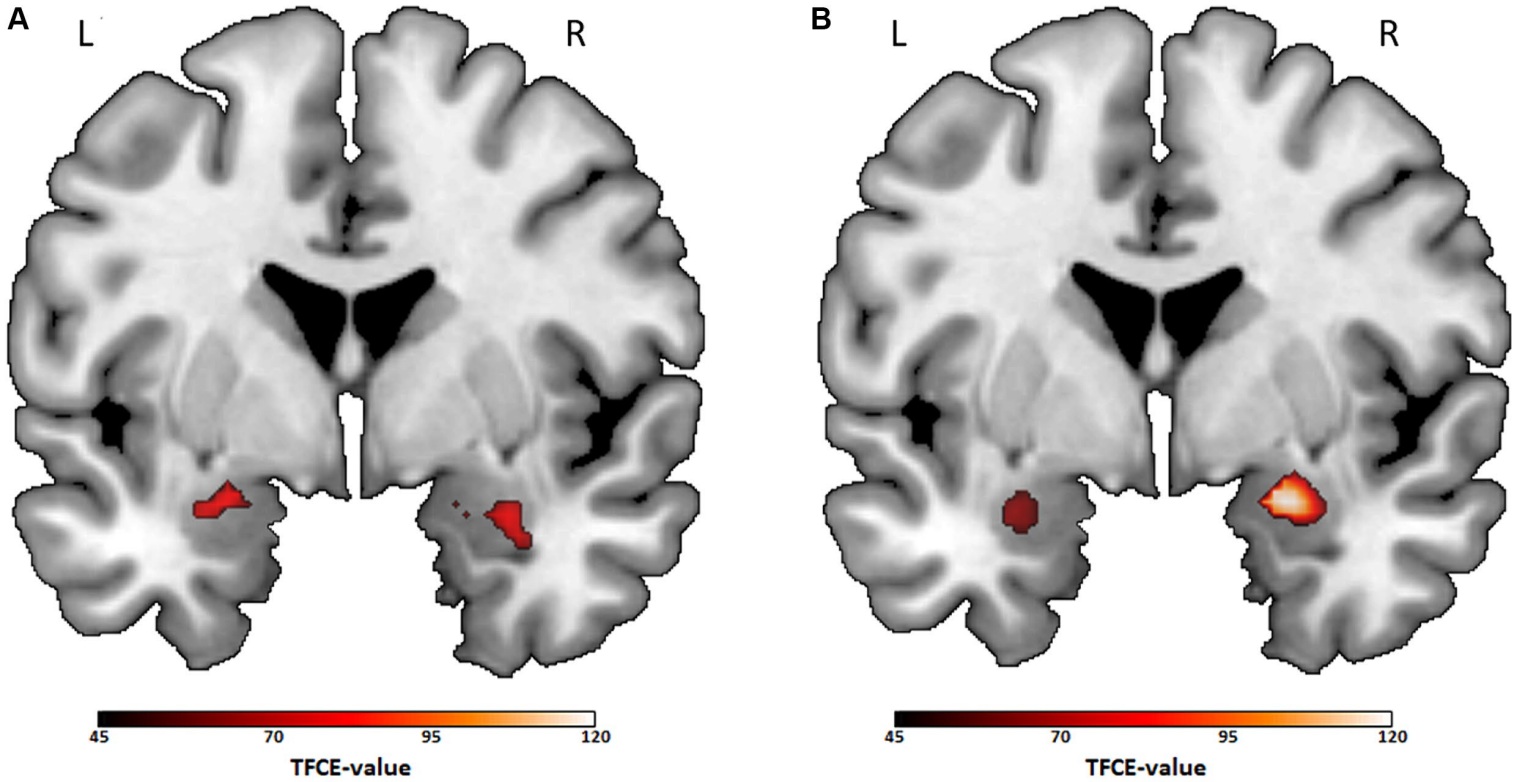
Bilateral amygdala activation for post-hoc comparisons between action vs. crime/thriller preference and comedy vs. crime/thriller preference. Note. Coronal views of the ROI analyses of the bilateral amygdala (*y* = 0) for two-sample *t*-tests including age, sex and time as covariates (*p*_TFCE-FWE_ < 0.05). Color bar: TFCE-value. **(A)** Contrast action preference > crime/thriller preference (mean cluster value at: left: *x* = −24, *y* = −6, *z* = −18, *k* = 91; right: *x* = 30, *y* = 2, *z* = −20, *k* = 73). **(B)** Contrast comedy preference > crime/thriller preference (mean cluster value at: left: *x* = −30, *y* = 2, *z* = −20, *k* = 80; right: *x* = 20, *y* = −4, *z* = −16, *k* = 104). L, left hemisphere; R, right hemisphere.

To compare movie genres directly, we took further investigations on genres with significant differences in limbic reactivity to emotional stimuli in previous analyses (crime/thriller, documentary, action and comedy). The same approach described above was followed for post-hoc ROI analyses. Since the study design does not allow direct comparisons between different genre preference groups (because some individuals preferred both compared genres), participants with a double preference for both compared genres were excluded for each comparison, resulting in new group sample sizes (see as follows). For the action (*n* = 43) vs. crime/thriller (*n* = 99) and comedy (*n* = 55) vs. crime/thriller comparison (*n* = 90), the ROI analysis for the amygdala showed higher bilateral activity for the action preference group (left: *x* = −24, *y* = −6, *z* = −18, *k* = 91, TFCE_(137)_ = 113.01, *t* = 3.71, *p*_TFCE-FWE_ = 0.013; right: *x* = 30, *y* = 2, *z* = −20, *k* = 73, TFCE_(137)_ = 86.55, *t* = 3.25, *p*_TFCE-FWE_ = 0.025, see Figure 2A) as well as for the comedy preference group (left: *x* = −30, *y* = 2, *z* = −20, *k* = 80, TFCE_(140)_ = 66.29, *t* = 2.86, *p*_TFCE-FWE_ = 0.027; right: *x* = 20, *y* = −4, *z* = −16, *k* = 104, TFCE_(140)_ = 119.81, *t* = 3.62, *p*_TFCE-FWE_ = 0.004, see Figure 2B), compared to the individuals of crime/thriller preference, respectively. For the action (*n* = 51) vs. documentary (*n* = 104) and comedy (*n* = 59) vs. documentary (*n* = 91) comparison, significant group differences for the right amygdala occurred, indicating higher activity within the action preference group (*x* = 32, *y* = 4, *z* = −26, *k* = 25, TFCE_(150)_ = 62.22, *t* = 3.41, *p*_TFCE-FWE_ = 0.017) and the comedy preference group (*x* = 30, *y* = 4, *z* = −28, *k* = 16, TFCE_(145)_ = 48.02, *t* = 2.79, *p*_TFCE-FWE_ = 0.033). For the crime/thriller (*n* = 77) vs. documentary (*n* = 74) and comedy (*n* = 66) vs. action (*n* = 45) comparison, no significant differences in amygdala activity occurred (all *p*_TFCE-FWE_ ≥ 0.189).

The ROI analyses of the NAcc showed higher right-sided activity for the action preference group than the crime/thriller preference group (*x* = 14, *y* = 12, *z* = −10, *k* = 6, TFCE_(137)_ = 13.84, *t* = 2.70, *p*_TFCE-FWE_ = 0.021). Individuals of comedy preference showed increased right-sided NAcc activity compared to individuals of the crime/thriller preference group (*x* = 14, *y* = 12, *z* = −10, *k* = 7, TFCE_(140)_ = 17.58, *t* = 2.87, *p*_TFCE-FWE_ = 0.009) and individuals of the documentary preference group (*x* = 12, *y* = 12, *z* = −10, *k* = 5, TFCE_(145)_ = 14.37, *t* = 2.78, *p*_TFCE-FWE_ = 0.009), respectively. For the action vs. documentary, documentary vs. crime/thriller, and comedy vs. action comparison, no significant differences in NAcc activity occurred (all *p*_TFCE-FWE_ ≥ 0.057).

## 4 Discussion

As the first of its kind, the present study investigated associations between movie genre preferences and brain activity in limbic and reward-related brain structures while processing negative emotional stimuli. As hypothesized, specific movie genres were linked to different patterns in amygdala activity during an fMRI paradigm using fearful and angry faces. Participants with a preference for action and those with a preference for comedy movies showed increased limbic reactivity. In contrast, a decreased limbic activity pattern was observed in participants with a preference for crime/thriller and participants with documentary preference, each compared to those without such preference. At the same time, the activity of the NAcc was higher for comedy preferences, whereas it was lower for crime/thriller and documentary preferences. Post-hoc comparisons of individuals who were either in favor of one or the other genre confirmed the contrary results for action and comedy in relation to crime/thriller and documentary. Furthermore, NAcc activity was higher in participants with an action preference and in participants with a comedy preference compared to those with a preference for crime/thriller, respectively. Additionally, NAcc activity was higher for comedy preference compared to documentary preference.

In order to find possible explanations for the different effect directions for action preference on the one hand and crime/thriller and documentary preference on the other hand, genre definitions shall be reviewed. While action and documentary could be positioned at opposite poles of various dimensions (e.g., fiction vs. nonfiction, emotional vs. unemotional), the demarcation between action and crime/thriller seems less clear. In fact, both genres share a joint emotional range, primarily characterized by eliciting primal and negatively valenced emotions like fear and anger by picturing dangerous, life-threatening, and violent scenes. Furthermore, excitation transfer is used as a stylistic tool to accumulate excitation throughout the plot to create an exaggerated excitation at its peak in movies of both genres. Hence, considering the significant overlap in emotional quality and intensity, the differences in limbic reactivity might seem unexpected.

Although similar emotional sensations are targeted in both action and crime/thriller genres, the underlying function might differ. According to Bartsch (2007a), genre preferences are not an expression of preferring primary emotions represented in or elicited by relevant movies but a result of gratifications derived from these primary cinematic emotions. However, each movie genre has the potential to entail various emotional gratifications, and the susceptibility to those gratifications varies inter-individually. Robinson et al. (2014), for example, identified three different types of horror fans based on whether they gain gratification predominantly from the emotional experience (“adrenaline junkies,” “white knucklers”) or from unraveling the mysterious story plot (“detectives”). Thus, for some genres, the emotional gratification potential predominantly lies in the experience of emotions and the accompanying arousal *per se*. On the other hand, emotions are more instrumental in other genres, e.g., to challenge the viewer’s coping capacities or to enhance story involvement.

Differences in brain activity during anger and fear processing could be linked to differences in how the mere experience of cinematic emotions and the accompanying arousal could function as gratification. Based on the decreased activity in the amygdala and the NAcc in individuals with crime/thriller preference - indicating lower emotional responsiveness and approach tendency to fearful and angry stimuli - it is likely that crime/thriller preference is associated with gratifications beyond the mere emotional experience. In their attempt to differentiate action and thriller, Irsigler et al. (2014) emphasize that the suspense in the latter is grounded in the viewer’s informational deficit and the mystery rather than in stress induced by fear or anger-triggering cinematic cues. Therefore, crime/thriller viewers may instead gain gratification in unraveling the mystery, even though fear and anger are prevalent. This aligns with Grodal (2017) who considers seeking to be the dominant emotion of crime/thriller. According to Grodal (2017), seeking is an emotion that motivates explorative behavior to find action-relevant cues in one’s environment. In crime/thriller movies, it is the driving force to resolve suspense and unravel the mystery. In fMRI studies using naturalistic movie-watching designs, suspense and anticipation of fear were associated with increased activity in attention networks and sensory areas rather than in emotional processing centers (Bezdek et al., 2013; Hudson et al., 2020), which is consistent with the idea of seeking as dominant emotion. Similarly, Salimpoor et al. (2011) found that dopamine is released in dissociable brain areas when listening to music, depending on whether the emotional peak is anticipated (state of tension/suspense) or actually happening. During anticipating, dopamine was primarily released in the dorsal striatum rather than the NAcc, in which dopamine was released right while emotional tension peaked. The dorsal striatum is known for its role in anticipatory processes and, therefore, for the state of seeking. As for crime/thriller preference, the diminished neuronal activity in response to fearful and angry stimuli in documentary viewers could be linked to a decreased susceptibility to find gratification in an emotional watching experience and, therefore, to the fact that documentary viewers prefer factual over emotional content.

For action movie preference, however, the enhanced limbic and NAcc reactivity to fearful and angry stimuli suggests that these emotional stimuli have a higher relevance and incentive potential and that the experience of these emotions is probably a primary source of gratification compared to crime/thriller. The fact that individuals with a high limbic response to fear and anger seek out movies that typically have a high density of scenes that represent and elicit these emotions suggests a relationship between reactivity to genre-specific emotions and preference in the case of action movies. Studies on drama (e.g., Oliver et al., 2000; Wagner et al., 2014) and horror movies (Sparks, 1991) have already demonstrated that a more intense experience of genre-related negative emotions leads to a more positive evaluation of these movies. Sparks (1991), for example, found that males with higher subjective and physical stress responses to a horror movie appreciated the movie the most. A recent study has also shown that higher fearfulness in horror movie scenes leads to greater enjoyment for viewers (Kiss et al., 2024). Other studies hint that the state of being emotionally aroused by art could be rewarding *per se* (e.g., music; Salimpoor et al., 2009) and thus function as a driving component of preference formation for content that affects one on an emotional level. Irsigler et al. (2014) support the idea that emotions in action movies are evoked for the sake of experiencing these emotions. According to the authors, suspense in action movies is stress-induced rather than a product of a narrative conflict, as in crime/thriller movies.

Interestingly, individuals with a preference for comedy movies showed a similar activity pattern in the amygdala and the NAcc as those with an action preference. However, unlike action movies, comedies are mainly known for portraying and eliciting positive emotions. The increased reward-associated brain activity to negative facial expressions in comedy preference might suggest a universal, valence-independent incentive value of affective facial expressions in this group. This would support the assumption that, as in the case of action movie preference, the genre-related gratification lies in the emotional movie experience *per se*.

Altogether, and following the thought that movie genres are preferred by those individuals who are the most susceptible to genre-specific gratification, it seems plausible that individuals who are more neurobiological prone to response and approach fear and anger (or affective stimuli in general) are those who prefer movies, which primarily rely on emotional arousal stemming from representing and eliciting these emotions. Conversely, it also makes sense that those with a low responsiveness and approach tendency to emotional stimuli, like fearful and angry faces, seek out movies involving viewers more on a cognitive than an emotional level, e.g., by picturing a mystery (crime/thriller) or factual information (documentary). However, inter-individual differences in brain reactivity to negative emotional stimuli could also be effects of repetitively watching movies of a particular genre. Unfortunately, our data do not allow conclusions about the direction of effects. An interaction of predispositions in emotional processing and genre exposure effects also seems plausible and should be investigated in future research.

Several studies dealt with the question of why media-elicited negative emotions are perceived as entertaining and are enjoyed by broad audiences. Although some first empirical attempts to unravel this question exist, even less is known about individual differences in preference for media genres varying in emotional profiles. The present study was the first to investigate brain activity correlates of movie genre preference with a particular account for preference-related differences in processing fearful and angry emotional stimuli. The results provide further insight into why individuals differ in their movie genre preferences. In this regard, the concept of movie-genre preferences as a function of susceptibility to genre-related emotional gratification potentials (Bartsch, 2007a), as well as a function of neuronal reactivity potentials (Grodal, 2017), finds support. However, this should be considered with caution unless the directions of effects are unclear. The present study contributes to a more precise demarcation between action and crime/thriller genres, often subsumed due to similarities in style and content. However, the contrary findings suggest that action and crime/thriller movies are sought out by people of different psychological conditions, probably because of differentiable gratification offerings, and therefore support a consideration as separate genres. Since research primarily has focused on movies of the fictional spectrum, little has been known about which factors are associated with preferences for non-fictional media content like documentaries. We were able to provide initial findings in this regard.

Some limitations should be acknowledged: First, the applied paradigm compared the neuronal processing of angry and fearful faces in contrast to shapes. Thus, we do not know whether the effects differ between fearful and angry faces. Moreover, the primary emotions of other genres, like disgust (horror), sadness (drama/romance), and happiness (comedy/romance), were not targeted, which could be the reason why the effects were limited to three or four of seven finally investigated genres. Second, we employed a well-established paradigm to investigate neuronal correlates of emotion processing. However, research under naturalistic and ecological valid neuroimaging conditions, e.g., by using movie content, is recommended. Third, it has not been proven whether all participants had a shared understanding of movie genre definitions. Fourth, only general but not genre-specific daily hours of movie watching were assessed, and therefore, it could not be controlled for genre-related exposure effects. Unfortunately, due to small preference numbers, no investigations on horror movie preference could be made, which is by far the best-studied movie genre in the literature (for a review, see Martin, 2019). Especially because of its fear-eliciting nature, it is highly interesting for future research following a method similar to the present study.

## 5 Conclusion

This current work is the first to investigate and successfully relate inter-individual differences in responsiveness to negative emotional stimuli on a brain level and movie genre preferences. Although more knowledge of effect direction is needed, the findings are an essential first step in understanding the key ingredients to produce the optimal kind and degree of stimulation and an enjoyable experience for recipients being in favor of one particular movie genre. Rather than “one size fits all,” the findings suggest that movie viewers form preferences aligned with their neuronal proneness to react to and approach certain cinematic stimuli. Therefore, future research should investigate the impact of media content exposure, compatible or incompatible with individual neuronal reactivity tendencies, on overall psychological well-being.

## Data availability statement

The raw data supporting the conclusions of this article will be made available by the authors on reasonable request, without undue reservation.

## Ethics statement

The studies involving humans were approved by ethic committee of Westfalen-Lippe. The studies were conducted in accordance with the local legislation and institutional requirements. The participants provided their written informed consent to participate in this study.

## Author contributions

EZ: Conceptualization, Data curation, Formal analysis, Methodology, Visualization, Writing – original draft. PK: Conceptualization, Data curation, Formal analysis, Methodology, Visualization, Writing – original draft. RH: Writing – review & editing. AK: Writing – review & editing. JS: Writing – review & editing. LP: Validation, Writing – review & editing. KS: Visualization, Writing – review & editing. MR: Writing – review & editing. VE: Data curation, Investigation, Writing – review & editing. TB: Data curation, Investigation, Writing – review & editing. MK: Data curation, Investigation, Writing – review & editing. KD: Data curation, Investigation, Writing – review & editing. EL: Data curation, Investigation, Writing – review & editing. JB: Investigation, Resources, Software, Writing – review & editing. UD: Funding acquisition, Investigation, Project administration, Supervision, Writing – review & editing. RR: Data curation, Formal analysis, Funding acquisition, Investigation, Methodology, Project administration, Supervision, Writing – original draft.
